# Commentary: Studies in Zebrafish Demonstrate That *CNNM2* and *NT5C2* Are Most Likely the Causal Genes at the Blood Pressure-Associated Locus on Human Chromosome 10q24.32

**DOI:** 10.3389/fcvm.2020.582101

**Published:** 2020-10-19

**Authors:** Daniela Sorriento, Guido Iaccarino

**Affiliations:** Department of Advanced Biomedical Sciences, Federico II University, Napoli, Italy

**Keywords:** animal models of disease, GWAS, high blood pressure (hypertension), transgenic mice, physiology

## Introduction

Hypertension is a critical health issue (1). The magnitude of the epidemiology of this condition is such that one third of the world population is affected by, with a high risk of developing cardiovascular complications, main cause of death (2). The pathogenetic mechanisms that trigger such condition are not completely understood due to the complex multifactorial etiology. Sex, age, ethnicity, an unbalanced diet (high salt, sugar, fat/cholesterol and alcohol intake, low potassium intake), environmental inputs (sedentary lifestyle, smoking, and chronic stress), comorbidities such as diabetes can predispose to essential hypertension. From a clinical point of view, the large variability in hypertensive phenotypes requires a thorough assessment of the patient in order to tailor personalized therapy. An important role is to be ascribed to the impact of genetic background, as testified by pivotal studies in twins and families (3, 4). Several putative hypertension-related genes have been identified, mainly affecting the renin-angiotensin-aldosterone system (5, 6). However, functional studies show that some of them have only mild or even no effects on the hypertensive phenotype (7). Recently, new bioinformatic tools and the Genome-wide association studies (GWAS) allowed a more accurate study of the genetic architecture of hypertension, identifying the associations between single nucleotide polymorphisms (SNPs) and specific hypertensive phenotypes (8–10). However, pre-clinical studies on the functional link between the identified genes and the development of hypertension are limited. Modeling in animals the genetics observed in humans represent an important step toward the proof of concepts of associative studies by GWAS.

## Historical Point of View

Since 1974, when Jaenisch and Mintz performed the first genetic manipulation in mice (11), these little mammals have become the unique tool to study human diseases. Similarities in the physiology and genetics of mice and humans have contributed to the popularity of this model. The real boost in mouse modeling derives from the seminal work of Mario Capecchi, Oliver Smithies and sir Martin Evans, awarded Nobel Prize in 2007 for their discoveries of specific gene manipulations in mice by the use of embryonic stem cells; since then, a fast and furious quest of technology has been able to perfect genetic engineering to generate novel phenotypes by inserting or deleting genes in specific tissues (12–14). In the last decades, the mouse models have been essential to increase the knowledge about the pathogenesis of several diseases and to identify novel targets for therapeutic purposes (15–21). Gene targeting in mice lead to several findings with important clinical implications, such as the protective role of bradykinin in diabetic nephropathy (22) and the dysfunction of CFTR gene in cystic fibrosis (23).

The deletion or overexpression of genes to understand relatively rare human genetic diseases that are caused by homozygous loss of gene function has become of routine use. Rather, modeling the much more common multifactorial diseases that have strong genetic and environmental causes is less easy (24).

## New Modeling

In this issue of Frontiers in Cardiovascular Medicine, Vishnolia et al. (25) provide an intriguing insight in validation of the results of a GWAS study in hypertension by performing a functional study in model that is novel to cardiovascular researchers: the zebrafish. This may appear very far from the commonly known pre-clinical models. This model has several advantages over the mouse in terms of maintenance, genetic manipulability, and the ability for high-throughput screening. In particular, zebrafish has become a recent new tool in the cardiovascular field (26–28) with promising implications in future translational studies. This model is particularly useful for cardiac studies thanks to the optical clarity of embryos that allows non-invasive *in vivo* imaging of fluorescently labeled cardiac genes during cardiac development without affecting the physiological setting of the disease. The transparent embryos also favors genetic manipulations through several techniques such as morpholino antisense oligonucleotide and CRISPR-Cas9 system (29–32). Furthermore, the zebrafish embryonic heart rate is much closer to the human heart rate than the mouse' (33). Using the zebrafish model, it has been demonstrated that MEIS2 is critical for proper heart tube formation and subsequent cardiac looping (34). The interaction of the genetic background with the environment can be facilitated by adding the appropriate perturbing agent to the water of the tank, which guarantee a uniform exposure of all individuals. Such aspect is essential to study multifactorial diseases, such as hypertension: gene targeting can identify modifiers genes that are turned on in response to environmental stimuli.

## Discussion

In their paper, Vishnolia et al. exploited the possibilities of this model to highlight that two genes, CNNM2 and NT5C2, identified by GWAS at the human 10q24.32 locus, among other putative genes (CYP17A1, BORCS7, AS3MT, CNNM2, and NT5C2), predispose to hypertension (25). They show that all genes encompassed in the human chromosome 10q24.32, CAD, and BP locus are highly conserved between zebrafish and humans. Using zebrafish morpholino-dependent knock-down and specific knock-out, they demonstrate that higher blood flow, increased arterial pulse, and elevated linear velocity are observed in larvae with genetic removal of CNNM2 and NT5C2. In this condition, ramipril, a commonly used antihypertensive drug, failed to increase blood flow. These data not only confirm the involvement of CNNM2 and NT5C2 genes in the development of hypertension but also propose the zebrafish model as a potential tool for anti-hypertensive drug screening, allowing to associate drugs treatment with a specific genetic mutation.

An interesting finding of this study is also the reciprocal regulation of these genes since the Authors show that NT5C2 knock-out induces downregulation of CNNM2 and conversely CNNM2 knock-out leads to a reduction of NT5C2 expression. Besides supporting the triggering role of these genes in hypertension, these findings also suggest a negative feedback regulation of hypertension-related genes that could, in part, explain a large amount of identified genetic variants without a strong association with the hypertensive phenotype.

The Authors also show that the knock-out models were associated with impaired renal function, high levels of renin, and significantly increased expression of the ren gene, suggesting that defects in the renin-angiotensin-aldosterone signaling pathway could be the culprit of NT5C2 and CNNM2 dependent increase of blood flow parameters. These findings are in line with the literature showing that impaired activation of the renin-angiotensin-aldosterone system (RAAS) is associated with hypertension (35, 36). Furthermore, they validate the use of this model that is also able to recapitulate in principle the target organ damage observed in hypertension.

Overall, this study has great translational relevance in the management of hypertensive phenotype by identifying two specific genes whose modulation is strongly associated and proposing a novel tool for anti-hypertensive drug screening.

The future direction for the use of this model let us envisage a new era of therapeutics for hypertension, that could take advantage of gene targeting. Of mice, zebrafishes and men is going to be the tale for hypertension research in the immediate future.

## Author Contributions

DS: writing first draft. GI: conceptualization, writing second draft. All authors contributed to the article and approved the submitted version.

## Conflict of Interest

The authors declare that the research was conducted in the absence of any commercial or financial relationships that could be construed as a potential conflict of interest.
